# Integrated Serum Metabolomics and Network Pharmacology to Reveal the Interventional Effects of Quzhi Decoction against Osteoarthritis Pain

**DOI:** 10.1155/2022/9116175

**Published:** 2022-08-12

**Authors:** Xiaoqing Shi, Peng Wu, Lishi Jie, Li Zhang, Jun Mao, Songjiang Yin

**Affiliations:** ^1^Affiliated Hospital of Nanjing University of Chinese Medicine, Nanjing 210029, China; ^2^Jiangsu Province Hospital of Chinese Medicine, Nanjing 210029, China; ^3^The Department of Traditional Chinese Osteopathy and Traumatology, First College of Clinical Medicine, Nanjing University of Chinese Medicine, Nanjing 210023, China

## Abstract

**Objectives:**

Chronic pain, the main symptom of knee osteoarthritis (OA), remains the primary reason for decreased functional capacity. Quzhi decoction, a TCM prescription, is effective in treating chronic pain in OA, but the potential mechanisms require further exploration.

**Methods:**

An anterior cruciate ligament transection (ACLT) rat model was established, and pain-like behavior was evaluated. Metabolomics analysis of serum samples was performed to identify differential metabolites, and network pharmacology was used to identify potential targets of Quzhi decoction for the treatment of OA. Finally, we constructed a comprehensive network of serum metabolomics and network pharmacology. At the same time, the obtained key targets were verified by molecular docking.

**Results:**

Quzhi decoction was shown to attenuate pain-like behavior and joint inflammation in OA rats. Through serum metabolomics, thirty potentially significant metabolites were found to be involved in the therapeutic effects of Quzhi decoction against OA pain. According to network pharmacology, 107 active drug components were matched with 115 disease targets, which was partly consistent with the metabolomics findings. Further analysis focused on 6 key targets, including CYP3A4, PLA2G4A, PTGS1, PTGS2, TYR, and ALOX5, and their associated core metabolites and pathways. Molecular docking results showed that the related targets had high affinity with the active pharmaceutical ingredients in Quzhi decoction.

**Conclusion:**

The effect of Quzhi decoction on OA pain may be related to the inhibition of joint inflammation, mainly through disturbing arachidonic acid metabolism, tyrosine metabolism, and leukotriene metabolism. Further systematic molecular biology experiments are needed to verify the accurate mechanism.

## 1. Introduction

Knee osteoarthritis (OA), a degenerative disease of the bones and joints, is a major cause of physical disability in older adults [1]. Pain is the main symptom of knee OA, and persistent chronic pain remains the primary reason for decreased functional capacity in patients [2]. Sensory nociceptors are mainly located in the synovium, which is recognized as a mediator or generator of pain. Progression in synovial thickening in KOA has been reported to correlate with increasing knee pain [3]; meanwhile, knee OA patients with pain are also known to have higher degrees of synovitis than patients without pain [4]. These facts further indicate that synovial inflammation could activate peripheral nociceptors, leading to the development of nociceptive pain in the OA joint.

To date, there is no effective treatment to relieve the pain of patients with knee OA. The long-term use of paracetamol and nonsteroidal anti-inflammatory drugs (NSAIDs) leads to adverse reactions, such as peptic ulcers and bleeding; thus, new treatment strategies are urgently needed [5]. Traditional Chinese medicine (TCM) has been demonstrated to improve clinical findings in degenerative diseases, and its curative effects are precise, safe, and reliable [6, 7]. Quzhi decoction, a TCM prescription from Yixue Zhongzhong Canxi Lu developed by Zhang Xi Chun in the Qing Dynasty, has been used to treat leg pain caused by liver deficiency. Recently, our previous clinical observations also suggested that the Quzhi decoction can relieve the pain and stiffness of KOA patients and improve physical function [8]. However, the mechanisms underlying these effects remain unclear.

Given that OA involves internal complex metabolic disorders [9], metabonomics for monitoring the dynamic changes of the metabolites pathology after herbal application is very useful. However, traditional metabolomics reflects only terminal variation of biological processes, when considered alone, they fail to explain the mechanism of Quzhi decoction resistance to OA pain. Network pharmacology appears to be an alternative system-level approach to explain the effects of drugs and their multicomponent, multitarget, and multipath synergistic mechanisms [10, 11]. Through the prediction of compound-target combinations and pathway analysis in network pharmacology, learning the endogenous metabolic processes becomes easier, including how these metabolites are produced, what their related pathways are, and which key proteins in Quzhi decoction exert its effects.

**Figure 1:**
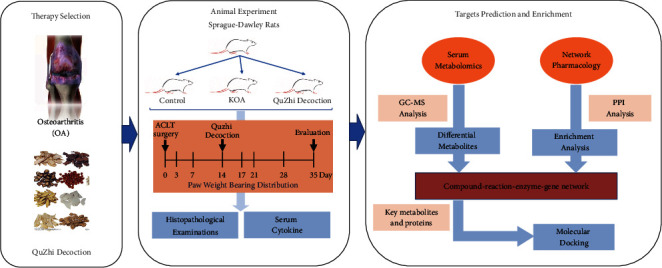
Quzhi decoction was used to treat osteoarthritis in the current article (therapy selection); the influence of Quzhi decoction on pain-like behavior and joint inflammation in knee OA rats (animal experiment); integrated serum metabolomics and network pharmacology to reveal the interventional effects of Quzhi decoction against osteoarthritis (targets prediction and enrichment).

Therefore, we first evaluated the effectiveness of Quzhi decoction in a rat model of OA pain. Furthermore, based on serum metabolomics and network pharmacology, this study established a comprehensive strategy to explore the key targets and mechanisms of Quzhi decoction in the treatment of OA. The detailed process of the current study is shown in Figure 1.

## 2. Methods and Materials

### 2.1. Animal Experiment

In brief, twenty-four Sprague Dawley (SD) rats, aged 6–8 weeks (provided by Nanjing Qing Long Shan Animal Technology Co. Ltd.), were randomly divided into three groups: sham, KOA, and KOA + Quzhi. The KOA model was constructed by anterior cruciate ligament transection (ACLT) surgery, as described previously [12], in the dextral knee joint. On the 14th day after ACLT surgery, the rats in the KOA + Quzhi group received an oral prescription with Quzhi decoction (shown in Table 1). All experiments were carried out in accordance with the National Institutes of Health Guide for the Care and Use of Laboratory Animals.

### 2.2. Measurement of Pain-Like Behavior

Changes in weight bearing were determined by an in-capacitance tester (SA-YLS-11A, SANS, Nanjing, China) following the procedures detailed by the manufacturer. The results were calculated as weight borne on the ipsilateral hindlimb as a percentage of total weight borne (dextral weight borne/(dextral weight borne + sinistral weight borne) × 100). At 0 ± 3°C temperature, in the cold plate (35150-001, Ugo Basil SLR, Italy) for measuring sensitivity to cold pain, three measurements were taken at an interval of 10 minutes and the average was obtained. Rats were tested on days 0, 3, 7, 14, 17, 21, 28, and 35 post-ACTG surgery.

### 2.3. Hematoxylin and Eosin Staining

Rat synovial tissue was taken and fixed with 10% neutral formalin. Next, it was soaked in EDTA and embedded in paraffin. Sections were HE-stained. The degree of synovitis was assessed using self-defined synovial pathological scores (see Supplementary Material 1 for details).

### 2.4. Quantification of Inflammatory Factors

The levels of IL-1*β*, IL-6, and MMP-1 in rat serum were estimated using a rat ELISA kit (FcMACS, Nanjing, China) according to the manufacturer's instructions.

### 2.5. Serum Sample Preparation

The serum samples from each group stored at 80°C were defrosted on ice. Then, 50 *μ*L was absorbed, and 200 *μ*L ice-cold methanol containing 12.5 *μ*g·mL^−1^ 1,2-C^13^ myristic acid was added, and the mixture was vortexed for 3 min. After centrifugation, 100 *μ*l supernatant was extracted at 4°C, 18000 r·min^−1^ for 10 min and dried at reduced pressure for 2 h, and pyridine solution with 30 *μ*L 10 mg·mL^−1^ methylamine hydrochloride was added, mixed for 5 min, and vibrated at 300 r·min^−1^ for 1.5 h. Then, 30 *μ*l BSTFA was added, vibrated at 300 r·min^−1^ for 0.5 h, and centrifuged for 10 min at 18000 r·min^−1^. From each sample, 5 *µ*L supernatant was taken and mixed well for the QC sample.

### 2.6. Chromatographic and Mass Spectrometry Conditions

TG-5MS capillary chromatography was performed on a capillary column with helium as the carrier gas. The volume flow rate was 1.2 mL·min^−1^, the inlet temperature was 250°C, the sample volume was 1 *μ*L, the split ratio was 20 : 1, and the temperature was programmed with an initial temperature of 60°C for 1 min and was then increased to 320°C at 20°C·min^−1^ and maintained for 5 min. Ion source temperature: 280°C, ion transport temperature: 250°C, ionization energy: 70 eV, mass spectrum scanning range: *m*/*z* 50∼500, and acquisition time: 3.5∼19.0 min. The instrument was equilibrated with 5 injections of QC, and then one injection of QC was added every 6 samples.

### 2.7. Data Processing Method

The chromatogram information collected by GC–MS was used for peak extraction and substance identification using MS-DIAL software and the NIST database. According to *p* < 0.05 and fold change (FC) >1.2 or<0.833, disease biomarkers of KOA were screened, and an FC test was performed on the data of the blank control group and the model group to determine FC >1.2 or <0.833. After the total ion flow normalization and Pareto calibration were carried out, the data were basically in line with the normal distribution. One-way ANOVA was conducted according to *p* < 0.05. The metabolites with statistically significant differences between the control group and the model group (FC >1.2 or <0.833) were selected to screen the metabolites of interest as disease biomarkers.

### 2.8. Network Pharmacology Construction

The active components of Quzhi decoction were identified with the criteria of oral bioavailability (OB) ≥30% and drug-likeness (DL) ≥0.18 through the Traditional Chinese Medicine Systems Pharmacology Database and Analysis Platform [13]. The targets related to KOA were uploaded from the OMIM [14], DisGeNET [15], GeneCards [16], DrugBank [17], and PharmGKB [18] databases to the UniProt database to obtain the corresponding gene information. Then, the target corresponding to the active ingredient of the drug and KOA is mapped to obtain the common targets of the drug and disease. The common targets were imported into Cytoscape software to construct a drug-drug active ingredient targets-disease-disease targets network.

Protein-protein interaction (PPI) networks were analyzed using the STRING database, and then the analytic results were visualized using Cytoscape. MCODE (Cytoscape plug-in) was used to carry out the module analysis, and the results were further analyzed for enrichment analysis by ClueGO in Cytoscape.

### 2.9. Key Proteins and Molecular Docking

Differential metabolites identified in metabolomics were imported into Cytoscape equipped with MetScape to obtain compound-reaction-enzyme-gene networks. This build was done to visualize the interactions between metabolites, pathways, enzymes, and genes. Key metabolites and proteins were identified by combining compound-reaction-enzyme-gene networks with genes and metabolic pathways.

We used this DS software to perform molecular docking analysis. The RCSB Protein Data Bank Database (https://www.rcsb.org/) was used to obtain the core target structures. With the Prepare Protein tool, the parameters set to the default values, and pretreatments such as dehydration, hydrogenation, and ring building included, we obtained the pretreated protein structures. The Define and Edit Binding Site tool was used to define the active center of the target molecule. The location and size of the active center were determined based on the positive ligand position for the protein as obtained from the PDB and related crystal information in the literature.

## 3. Results

### 3.1. Quzhi Decoction Attenuates Pain-Like Behavior and Joint Inflammation in KOA Rats

**Figure 2:**
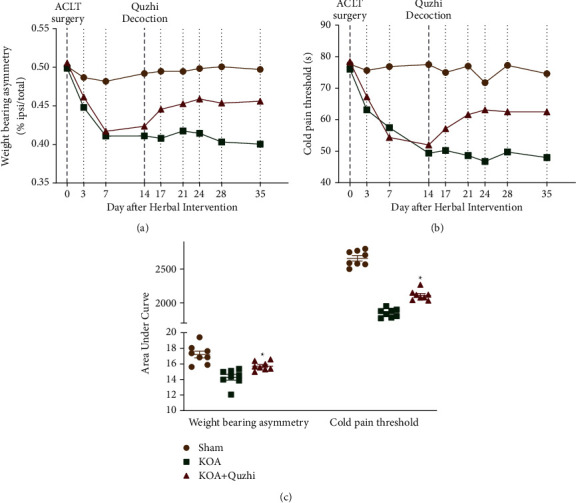
Quzhi decoction attenuates pain-like behavior in knee OA rats. At days 0, 3, 7, 14, 17, 21, 28, and 35 post-ACLT surgery, weight-bearing asymmetry was determined by an in-capacitance tester. (a) The results were calculated as weight borne on the ipsilateral hindlimb as a percentage of total weight borne (dextral weight borne/(dextral weight borne + sinistral weight borne) × 100); (b) cold pain threshold testing was performed on a cold plate (35150-001, Ugo Basil SLR, Italy) with a temperature of 0 ± 3°C; (c) the area under the curve of weight-bearing asymmetry or cold pain threshold from day 0 to 35 was also calculated. The results are expressed as the mean ± SEM. *N* = 8, *∗p* < 0.05. OA osteoarthritis.

In our current article, pain-like behavior was measured by the weight-bearing test and cold pain sensitivity. As expected, a reduction in spontaneous weight-bearing on the affected joint, indicative of joint pain, was observed after ACLT surgery. In addition, an attenuated response was detected in both the weight-bearing test and the cold pain sensitivity test by pharmacological application of the Quzhi decoction, as shown in Figure 2, suggesting that Quzhi decoction indeed attenuates pain-like behavior in KOA rats.

**Figure 3:**
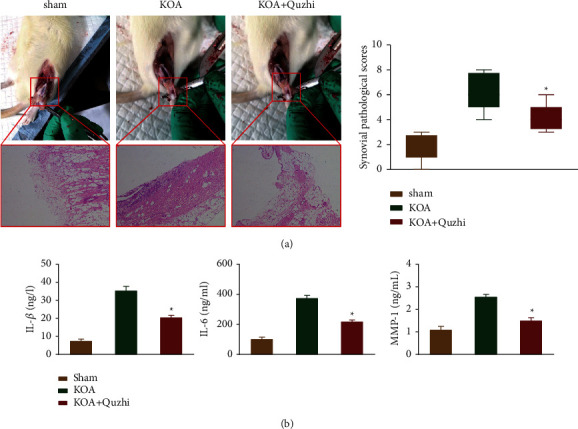
Quzhi decoction improves synovial inflammation in knee OA rats. (a) Representative images of histopathological HE staining of the synovium in rats are displayed (×200), and self-defined synovial pathological scores were calculated. (b) IL-1*β*, IL-6 and MMP-1 serum concentrations were also calculated by immunoassay, and the results are expressed as the mean ± SEM. *n* = 8, *∗p* < 0.05. OA: osteoarthritis, IL: interleukin, MMP matrix: metalloproteinase.

Some studies have attributed the pain-like behavior in animal models to joint inflammation; therefore, the histological pathological changes were evaluated in the current article. Our results suggested that severe synovial inflammation was found in KOA rats, which was markedly inhibited by the pharmacological application of Quzhi decoction (Figure 3(a)). In addition, the upregulation of serum inflammatory and catabolic factors was observed after ALCT surgery. Similarly, these responses in KOA rats significantly attenuated the prescription of herbal medicine (Figure 3(b)).

### 3.2. Identification of Different Metabolites after Quzhi Decoction Treatment in KOA Rats

**Figure 4:**
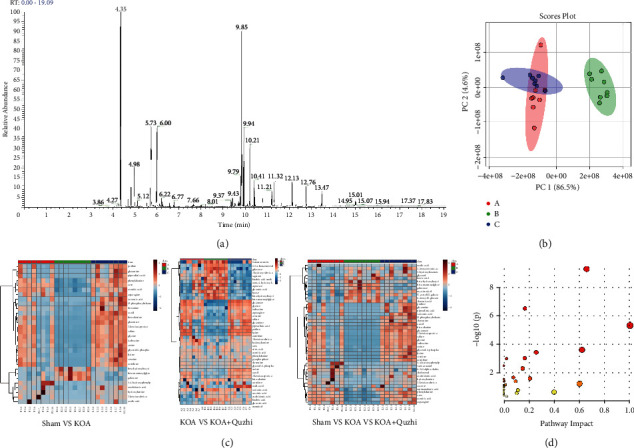
The identification of different metabolites after Quzhi decoction treatment in KOA rats. (a) Total ion chromatogram (TIC) of the methanol extract acquired by GS-MS; (b) PLSDA of metabolites in the KOA rats. Each point in the figure represents a sample, and all the sample points are distributed in the circular region. *N* = 8; (c) heatmap of identified differential metabolites among the sham, KOA and KOA + Quzhi groups; (d) bubble chart of metabolic pathways from significant metabolites. Node size is based on impact values, and node color is based on −log10 (*p*) values.

A total ion flow diagram of typical GC-MS analysis of serum samples from all groups is shown in Figure 4(a). A total of 98 compounds were identified using the MS-DIAL and NIST databases, and the peak height, retention time, and compound names were obtained. The peak height of the internal standard substance in all samples was monitored, and its RSD was calculated to be 11.2364%, indicating the stability of the instrument.

The metabolite name, sample number, and peak height information obtained by MS-DIAL were imported into MetaboAnalyst for principal component analysis (PCA), and the results are shown in Figure 4(b). There was a significant difference between the control and model groups, suggesting that there were significant differences in serum metabolites between the control group and the KOA rats. The “Quzhi Decoction” group was significantly separated from the model group and was closer to the control group, suggesting that medication could regulate the metabolites in rats to a normal level to a certain extent, thus exerting anti-inflammatory effects. Finally, 43 metabolites with significant differences between the control and model groups were identified, namely, disease biomarkers of knee OA. Then, cluster analysis was conducted on the normalized data of differential metabolites. In total, 44 metabolites with significant differences between the “Quzhi Decoction” group and the model group were identified. Among them, there were 30 different metabolites in easy-layer callback (Figure 4(c)). Therefore, it is speculated that Quzhi decoction application may play a role in the treatment of KOA by regulating the levels of these 30 metabolites and related metabolic pathways. The 30 different metabolites were imported into MetaboAnalyst for enrichment analysis of metabolic pathways, and 28 metabolic pathways were obtained (details in Figure 4(d) and Supplementary Material 2).

### 3.3. Mechanism Research of Quzhi Decoction Based on Network Pharmacology

**Figure 5:**
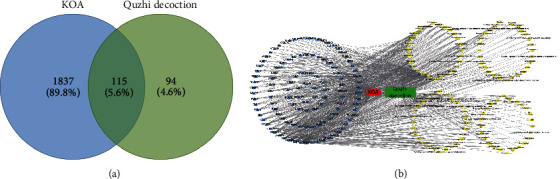
Network pharmacology construction. (a) Venn diagram showing the intersection of action targets of Quzhi decoction and knee OA targets; (b) Quzhi decoction treatment of the knee OA active ingredient—common targets network. The blue triangle represents the common targets, and the yellow rectangle represents the active components of Quzhi decoction. OA osteoarthritis.

To further reveal the mechanism of action of Quzhi decoction in the treatment of KOA, network pharmacology was used to study the targets and related pathways, providing new ideas for the development of drugs for treating KOA. A total of 1,952 knee OA gene targets were researched, which were mapped with two hundred nine active drug targets to obtain 115 common targets. The Venn diagram is shown in Figure 5(a), and the common target information is shown in Supplementary Material 3. Meanwhile, 107 active drug components were matched with 115 disease targets and a drug target-disease intersection network (Figure 5(b)).

**Figure 6:**
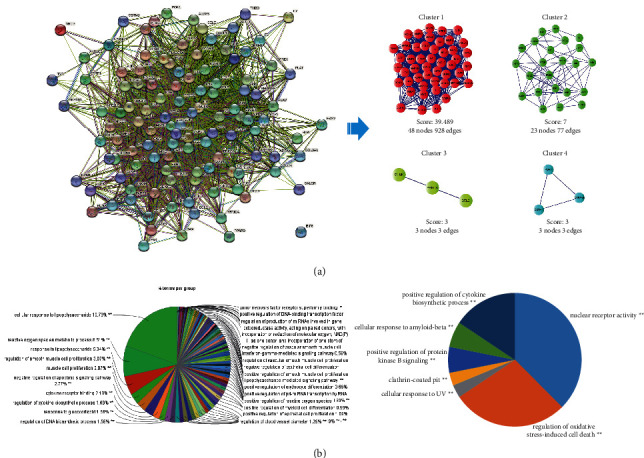
PPI network and GO enrichment analysis. (a) The PPI network of Quzhi decoction treatment on knee OA. Module analysis was carried out by MCODE. (b) GO enrichment analysis of Cluster 1 and Cluster 2 by ClueGO.

According to the PPI networks, 115 nodes and 2294 edges were identified. Additionally, two modules were selected using MCODE analysis (Figure 6(a)), in which Cluster 1 consisted of 48 nodes and 928 edges and Cluster 2 consisted of 23 nodes and 77 edges. We performed GO enrichment analyses by ClueGO (Figure 6(b)). The top terms in Cluster 1 were cellular response to lipopolysaccharide, reactive oxygen species metabolic process, response to lipopolysaccharide, muscle cell proliferation, and negative regulation of apoptotic signaling pathway, while the GO enrichments in Cluster 2 were nuclear receptor activity, regulation of oxidative stress-induced cell death, regulation of endothelial cell proliferation, and positive regulation of cytokine biosynthetic process.

### 3.4. Integrated Analysis of Serum Metabolomics and Network Pharmacology

**Figure 7:**
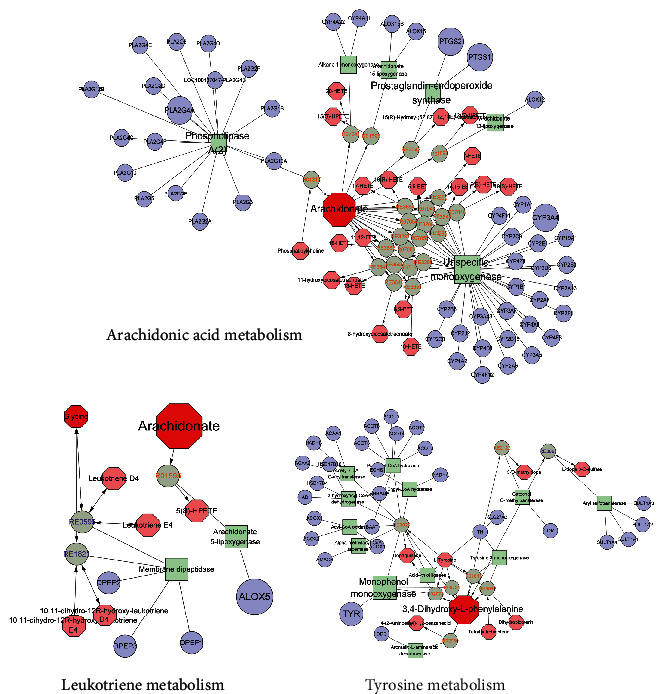
Compound-reaction-enzyme-gene networks of the key metabolites and targets. The red hexagons, gray diamonds, green round rectangles, and purple circles represent the active compounds, reactions, proteins, and genes, respectively. The key metabolites, proteins, and genes were magnified.

In order to understand the against OA mechanism of Quzhi decoction, we constructed an interaction network based on serum metabolomics and network pharmacology. MetScape (Cytoscape plug-in) was used to collect the compound-reaction-enzyme-gene networks. The potential targets identified in network pharmacology and genes in MetScape analysis were overlapped to show the intersection genes. Finally, we found 6 key targets, including CYP3A4, PLA2G4A, PTGS1, PTGS2, TYR, and ALOX5 (Table 2). The related key metabolites were arachidonate, glycine, and 3,4-dihydroxy-L-phenylalanine. The affected pathways were arachidonic acid metabolism, tyrosine metabolism, and leukotriene metabolism (Figure 7). These pathways may play essential roles in the therapeutic effect of Quzhi decoction on OA.

### 3.5. Molecular Docking

**Figure 8:**
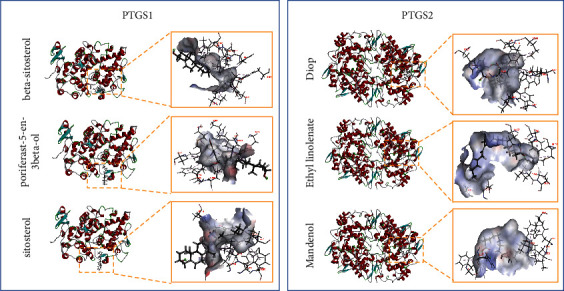
The 3D interaction diagrams of active drug components and the PTGS1 and PTGS2 targets.

In order to further study the possibility of interaction between Quzhi decoction and key targets, molecular docking study was carried out. The key targets CYP3A4, PLA2G4A, PTGS1, and PTGS2 could be analyzed by molecular docking after searching the RCSB Protein Data Bank database.  Figure 8 shows the 3D docking poses of luteolin with the core targets PTGS1 and PTGS2. Successful docking indicates that the active component may act on the relevant target and is a potential effective component with certain pharmacodynamics. However, the specific pharmacodynamic effects need to be verified by further *in vivo* and *in vitro* experiments.

## 4. Discussion

Chronic joint pain is the major problem associated with knee OA and current prescriptions widely used in clinical settings mainly focus on pain management [2]. Quzhi decoction, a TCM prescription from Yixue Zhongzhong Canxi Lu, consists of six Chinese herbs (Shanzhuyu, Zhimu, Danggui, Danshen, Ruxiang, and Moyao). Prior studies revealed that these Chinese herbs can relieve clinical symptoms and improve the quality of life of patients with knee OA [19, 20]. In the current study, we found that Quzhi decoction significantly improved cold pain sensitivity and/or weight-bearing discrepancy in OA rats. Moreover, accumulating evidence has demonstrated that extensive synovial inflammation, characterized by the secretion of catabolic and proinflammatory mediators, contributes to the development and maintenance of hyperalgesia and pain-like behavior in knee OA [3, 21]. As expected, we also found that severe synovial inflammation was markedly attenuated by the pharmacological application of Quzhi decoction. These facts suggested that the control of joint pain by Quzhi decoction may be linked to the inhibition of joint inflammation in rats with knee OA.

To reveal possible further mechanisms of Quzhi decoction against OA pain, serum metabolomics and network pharmacology combinations were constructed in our paper. In total, in easy-layer callback caused by Quzhi decoction, 30 significant metabolites were identified. However, given the complexity and heterogeneity of metabolomics, network pharmacology was used to screen the possible metabolites of Quzhi decoction in our paper. As a result, 115 disease targets in OA were screened. The targets were mainly enriched in ‘cellular response to lipopolysaccharide', ‘reactive oxygen species metabolic process,' and ‘nuclear receptor activity'. Lipopolysaccharide (LPS), also known as endotoxin, has been shown to contribute to low-grade inflammation in a number of clinical conditions, including cardio-metabolic dysfunction, acceleration of atherosclerosis, and diabetes [21, 22]. As a low-grade inflammatory condition, knee OA severity and joint inflammation are also associated with elevated levels of LPS [23, 24]. Pathologically, OA-related synoviocytes significantly express functional TLR4, and activation of the TLR4 response to LPS (a TLR4 agonist) triggers an inflammatory response [25], leading to the development of synovial inflammation and joint pain. Meanwhile, the local inflammatory response can contribute to an imbalance of the oxidative and antioxidant systems. Inflammatory mediators, including IL-1*β* and IL-6, are highly upregulated in OA joints and induce reactive oxygen species (ROS) production and the expression of matrix-degrading proteases, leading to joint dysfunction [26, 27]. ROS and inflammation are interdependent, each being the ideal target for the treatment of OA [28]. These findings further explain the pharmacological inhibition of synovial inflammation by Quzhi decoction.

By combining serum metabolomics with network pharmacology, six key targets were found, and to further investigate the possibility of interaction, we applied molecular docking studies. As expected, the key targets CYP3A4, PLA2G4A, PTGS1, and PTGS2 could be analyzed by molecular docking, which indicates that the active component may act on the relevant target. Prostaglandin-endoperoxide synthase (PTGS), also known as cyclooxygenase (COX), is the key enzyme in the synthesis of prostaglandin, including PTGS1 and inducible PTGS2 [29]. Previous studies have demonstrated that PTGS1 and PTGS2 are both highly expressed in the joints of patients with OA, which could provide drug targets for treating arthritis [30, 31]. Emerging evidence indicates that specific inhibitors of PTGS2 might offer more gastrointestinal safety than NSAIDs, which regulate both PTGS1 and PTGS2. However, it has also been reported that nonselective NSAIDs, such as naproxen, are not associated with cardiovascular risk [32]. The long half-life of Quzhi decoction and the complete and persistent inhibition of PTGS1 activity provide optimal conditions for OA therapy. In the current article, the anti-inflammatory and analgesic effects of Quzhi decoction may be explained by the capacity of these agents to disturb the two key targets. Apart from PTGS, the inflammation-related genes CYP3A4 and PLA2G4A, both involved in prostaglandin synthesis, have oxidation functions and play important roles in the metabolism of many endogenous and exogenous compounds [33, 34]. In addition, as the most abundant metabolic enzyme in the human liver, CYP3A4 regulates the drug metabolism of Quzhi decoction.

## 5. Conclusion

In this study, we first identified that Quzhi decoction was capable of improving pain-like behavior and joint inflammation in KOA rats. Subsequently, serum metabolomics and network pharmacology-integrated analyses revealed 6 targets as well as related metabolites and pathways. The key targets CYP3A4, PLA2G4A, PTGS1, and PTGS2 were further validated by molecular docking. These facts suggested that the control of OA pain by Quzhi decoction may be attributed to the inhibition of joint inflammation, mainly through disturbing prostaglandin metabolism. Further systematic molecular biology experiments are needed to verify the accurate mechanisms.

## Figures and Tables

**Table 1:** Herbs in Quzhi decoction.

Chinese medicine name	Latin name	Ratio by weight	Part used
Shanzhuyu	Cornus Officinalis sieb.et Zucc	30	Flesh
Zhimu	*Anemarrhenae Rhizoma*	18	Root and rhizome
Danggui	Angelicae Sinensis Radix	9	Root and rhizome
Danshen	Radix Salviae Miltiorrhizae	9	Root
Ruxiang	Boswellia Frereana	9	Resin
Moyao	Commiphora Molmol	9	Resin

**Table 2:** Information on key targets, metabolites, and pathways.

Related pathways	Key targets	Key metabolites
Arachidonic acid metabolism	CYP3A4, PLA2G4A, PTGS1, PTGS2	Arachidonic acid
Tyrosine metabolism	TYR	Arachidonic acid, glycine
Leukotriene metabolism	ALOX5	3, 4-Dihydroxy-L-phenylalanine

## Data Availability

All data included in this study are available upon request by contact with the corresponding author.

## Supplementary Materials

Supplementary Materials Supplementary Material 1: self-defined synovial pathological scores. Supplementary Material 2: serum metabolites and metabolic pathway. Supplementary Material 3: the common gene of Quzhi decoction in the treatment of OA.

## References

[1] Bijlsma J. W., Berenbaum F., Lafeber F. P. (2011). Osteoarthritis: an update with relevance for clinical practice. *The Lancet*.

[2] Schaible H. G. (2018). Osteoarthritis pain. Recent advances and controversies. *Current Opinion in Supportive and Palliative Care*.

[3] Shu C. C., Zaki S., Ravi V., Schiavinato A., Smith M. M., Little C. B. (2020). The relationship between synovial inflammation, structural pathology, and pain in post-traumatic osteoarthritis: differential effect of stem cell and hyaluronan treatment. *Arthritis Research and Therapy*.

[4] Ostojic M., Ostojic M., Prlic J., Soljic V. (2019). Correlation of anxiety and chronic pain to grade of synovitis in patients with knee osteoarthritis. *Psychiatria Danubina*.

[5] Pelletier J. P., Martel-Pelletier J., Rannou F., Cooper C. (2016). Efficacy and safety of oral NSAIDs and analgesics in the management of osteoarthritis: evidence from real-life setting trials and surveys. *Seminars in Arthritis and Rheumatism*.

[6] Lo P. C., Lin F. C., Tsai Y. C., Lin S. K. (2019). Traditional Chinese medicine therapy reduces the risk of total knee replacement in patients with knee osteoarthritis. *Medicine (Baltimore)*.

[7] Wang L., Zhang X. F., Zhang X. (2020). Evaluation of the therapeutic effect of traditional Chinese medicine on osteoarthritis: a systematic review and meta-analysis. *Pain Research and Management*.

[8] Shi X. Q., Zhang L., Xing R. L., Mao J., Zhang N. S., Wang P. M. (2021). Clinical experience in treating synovitis of knee osteoarthritis from professor Wang PM. *Journal of Basic Chinese Medicine*.

[9] Courties A., Berenbaum F., Sellam J. (2019). The phenotypic approach to osteoarthritis: a look at metabolic syndrome-associated osteoarthritis. *Joint Bone Spine*.

[10] Zhu N., Hou J., Ma G., Liu J. (2019). Network pharmacology identifies the mechanisms of action of shaoyao gancao decoction in the treatment of osteoarthritis. *Medical Science Monitor*.

[11] Jian G. H., Su B., Zhou W., Xiong H. (2020). Application of network pharmacology and molecular docking to elucidate the potential mechanism of eucommia ulmoides-radix achyranthis bidentatae against osteoarthritis. *BioData Mining*.

[12] Wu P., Huang Z., Shan J. (2020). Interventional effects of the direct application of “Sanse powder” on knee osteoarthritis in rats as determined from lipidomics via UPLC-Q-exactive orbitrap MS. *Chinese Medicine*.

[13] Xu X., Zhang W., Huang C. (2012). A novel chemometric method for the prediction of human oral bioavailability. *International Journal of Molecular Sciences*.

[14] Amberger J. S., Bocchini C. A., Schiettecatte F., Scott A. F., Hamosh A. (2015). OMIM.org: online Mendelian Inheritance in Man (OMIM®), an online catalog of human genes and genetic disorders. *Nucleic Acids Research*.

[15] Piñero J., Bravo A., Queralt-Rosinach N. (2017). DisGeNET: a comprehensive platform integrating information on human disease-associated genes and variants. *Nucleic Acids Research*.

[16] Stelzer G., Rosen N., Plaschkes I. (2016). The GeneCards suite: from gene data mining to disease genome sequence analyses. *Current Protocols in Bioinformatics*.

[17] Wishart D. S., Feunang Y. D., Guo A. C. (2018). Drugbank 5.0: a major update to the drugbank database for 2018. *Nucleic Acids Research*.

[18] Barbarino J. M., Whirl‐Carrillo M., Altman R. B., Klein T. E. (2018). PharmGKB: a worldwide resource for pharmacogenomic information. *Wiley interdisciplinary reviews. Systems biology and medicine*.

[19] Luo Y., Wang C. Z., Sawadogo R., Tan T., Yuan C. S. (2020). Effects of herbal medicines on pain management. *The American Journal of Chinese Medicine*.

[20] Chen B., Zhan H., Marszalek J. (2016). Traditional Chinese medications for knee osteoarthritis pain: a meta-analysis of randomized controlled trials. *The American Journal of Chinese Medicine*.

[21] Tilg H., Moschen A. R. (2014). Microbiota and diabetes: an evolving relationship. *Gut*.

[22] Harte A. L., da Silva N. F., Creely S. J. (2010). Elevated endotoxin levels in non-alcoholic fatty liver disease. *Journal of Inflammation*.

[23] Huang Z., Kraus V. B. (2016). Does lipopolysaccharide-mediated inflammation have a role in OA?. *Nature Reviews Rheumatology*.

[24] Huang Z. Y., Stabler T., Pei F., Kraus V. (2016). Both systemic and local lipopolysaccharide (LPS) burden are associated with knee OA severity and inflammation. *Osteoarthritis and Cartilage*.

[25] Wu R., Long L., Chen Q. (2017). Effects of Tim-3 silencing on the viability of fibroblast-like synoviocytes and lipopolysaccharide-induced inflammatory reactions. *Experimental and Therapeutic Medicine*.

[26] Ansari M. Y., Ahmad N., Haqqi T. M. (2020). Oxidative stress and inflammation in osteoarthritis pathogenesis: role of polyphenols. *Biomedicine and Pharmacotherapy*.

[27] Qu Z. A., Ma X. J., Huang S. B. (2020). SIRT2 inhibits oxidative stress and inflammatory response in diabetic osteoarthritis. *European Review for Medical and Pharmacological Sciences*.

[28] Srivastava S., Saksena A. K., Khattri S., Kumar S., Dagur R. S. (2016). Curcuma longa extract reduces inflammatory and oxidative stress biomarkers in osteoarthritis of knee: a four-month, double-blind, randomized, placebo-controlled trial. *Inflammopharmacology*.

[29] Agúndez J. A., Blanca M., Cornejo-Garcia J. A., Garcia-Martin E. (2015). Pharmacogenomics of cyclooxygenases. *Pharmacogenomics*.

[30] Li Z., Wang Q., Chen G. (2018). Integration of gene expression profile data to screen and verify hub genes involved in osteoarthritis. *BioMed Research International*.

[31] Zhang X., Bu Y., Zhu B. (2018). Global transcriptome analysis to identify critical genes involved in the pathology of osteoarthritis. *Bone and Joint Research*.

[32] Pepine C. J., Gurbel P. A. (2017). Cardiovascular safety of NSAIDs: additional insights after PRECISION and point of view. *Clinical Cardiology*.

[33] Xiao P., Zhu X., Sun J. (2021). LncRNA NEAT1 regulates chondrocyte proliferation and apoptosis via targeting miR-543/PLA2G4A axis. *Human Cell*.

[34] Shi Y. Y., Li Y. Q., Xie X. (2020). Homotherapy for heteropathy active components and mechanisms of Qiang-Huo-Sheng-Shi decoction for treatment of rheumatoid arthritis and osteoarthritis. *Computational Biology and Chemistry*.

