# Supplementary material for: Integrated Serum Metabolomics and Network Pharmacology to Reveal the Interventional Effects of Quzhi Decoction against Osteoarthritis Pain
Source: Int J Anal Chem. 2022 Aug 12;2022:9116175. doi: 10.1155/2022/9116175 (PMC9391123; doi:10.1155/2022/9116175)
Supplement: Supplementary Materials — Supplementary Material 1: self-defined synovial pathological scores. Supplementary Material 2: serum metabolites and metabolic pathway. Supplementary Material 3: the common gene of Quzhi decoction in the treatment of OA. [file 9116175.f1.zip › Supplementary Material 1.pdf]

Self-defined synovial pathological scores

H&E stained sections of synovium from both joints of all rats, were coded and scored blind by three observers. The scores ranged from 0 to 3 on the tissue criteria outlined in following table 1. All sections were assessed by both observers to avoid bias from interobserver variability, and five randomly selected areas were counted per section at each observation.

Table 1: Non-parametric scoring of rat synovial histopathology

Criteria	Score	Observation
Intimal hyperplasia	0	1-2 layers, only
	1	3-4 layers, focal
	2	2-5 layers, focal
	3	3-5 layers, diffuse
Lymphocytic/plasmocytic infiltration	0	None
	1	One focus of infiltration
	2	foci of infiltration
	3	Diffuse infiltration or >5 foci
Vascularity	0	0-2 vascular elements per 100 field
	1	3-4 vascular elements per 100 field
	2	5-8 vascular elements per 100 field
	3	>8 vascular elements per 100 field
