# Supplementary material for: Integrated Serum Metabolomics and Network Pharmacology to Reveal the Interventional Effects of Quzhi Decoction against Osteoarthritis Pain
Source: Int J Anal Chem. 2022 Aug 12;2022:9116175. doi: 10.1155/2022/9116175 (PMC9391123; doi:10.1155/2022/9116175)
Supplement: Supplementary Materials — Supplementary Material 1: self-defined synovial pathological scores. Supplementary Material 2: serum metabolites and metabolic pathway. Supplementary Material 3: the common gene of Quzhi decoction in the treatment of OA. [file 9116175.f1.zip › Supplementary Material 2.pdf]

Serum Metabolites

Sham VS KOA	KOA VS KOA+Quzhi	Sham VS KOA VS KOA+Quzhi
2-deoxytetronic acid	1-2-4-benzenetriol	2-ketoisocaproic acid
2-hydroxybutanoic acid	1-monostearin	2-ketoisovaleric acid
2-ketoisocaproic acid	2-hydroxyvaleric acid	3-(4-hydroxyphenyl)propionic acid
2-ketoisovaleric acid	2-ketoisocaproic acid	aconitic acid
3-(4-hydroxyphenyl)propionic	2-ketoisovaleric acid	arachidonic acid
6-deoxy-D-glucose	3-(4-hydroxyphenyl)propionic	asparagine
8-24(5-alpha)-cholestadien-3-beta-ol	aconitic acid	beta-alanine
aconitic acid	arachidonic acid	beta-hydroxybutyric acid
aminomalonic acid	asparagine	beta-mannosylglycerate
arachidonic acid	aspartate	creatine
asparagine	beta-alanine	galactose
beta-alanine	beta-hydroxybutyric acid	glutamate
beta-hydroxybutyric acid	beta-mannosylglycerate	glutamine
beta-mannosylglycerate	carnitine	glycerol-1-phosphate
cholesterol	citric acid	glycine
creatine	creatine	hydroxylamine
galactose	galactose	isoleucine
glutamate	gluconic acid	lysine
glutamine	glutamate	O-phosphorylethanolamine
glycerol	glutamic acid	ornithine
glycerol-1-phosphate	glutamine	oxalic acid
glycine	glycerol-1-phosphate	phenylalanine
hydroxylamine	glycine	pipecolinic acid
isofucostanol	hydroxylamine	proline
isoleucine	isoleucine	serine
lauric acid	linoleic acid	tartronic acid
lysine	linoleic acid methyl ester	threonine
myo-inositol	lysine	uracil
N-acetyl-D-galactosamine	lyxose	urea
O-phosphorylethanolamine	mannitol	valine
ornithine	O-phosphorylethanolamine	
oxalic acid	ornithine	
phenylalanine	oxalic acid	
pipecolinic acid	pantothenic acid	
proline	phenylalanine	
serine	pipecolinic acid	
sucrose	proline	
tartronic acid	pyrophosphate	
threonic acid	serine	
threonine	succinic acid	
uracil	tagatose	
urea	tartronic acid	
valine	threonine	
	trans-4-hydroxy-L-proline	
	uracil	
	urea	
	valine	

Metabolic pathway

	Total	Expected	Hits	Raw p	-lg(p)	Holm adjust	FDR	Impact
Glycine, seri	34	0.3838	8	5.96E-10	9.2251	5E-08	5E-08	0.65596
Arachidonic	48	0.54183	7	3.27E-07	6.4857	0.0000271	0.0000137	0.16667
Phenylalanin	4	0.045153	3	4.75E-06	5.3229	0.0003898	0.0001331	1
Phenylalanin	12	0.13546	3	0.0002472	3.6069	0.020024	0.0051914	0.61904
Cysteine and	33	0.37251	4	0.0003727	3.4287	0.029814	0.0062609	0.25594
Tyrosine me	42	0.4741	4	0.0009571	3.019	0.07561	0.01244	0.16435
Pantothenate	19	0.21448	3	0.0010367	2.9844	0.08086	0.01244	0.02143
Valine, leuci	8	0.090305	2	0.0032285	2.491	0.24859	0.033899	0
Glyoxylate a	32	0.36122	3	0.0048437	2.3148	0.36812	0.045208	0.14815
Citrate cycle	20	0.22576	2	0.020229	1.694	1	0.16992	0.07615
Pyruvate me	22	0.24834	2	0.02427	1.6149	1	0.18484	0.20684
Propanoate r	23	0.25963	2	0.026406	1.5783	1	0.18484	0
Alanine, asp	28	0.31607	2	0.038169	1.4183	1	0.22901	0.0024
Glutathione r	28	0.31607	2	0.038169	1.4183	1	0.22901	0.09216
Synthesis an	5	0.056441	1	0.055253	1.2576	1	0.30942	0.6
Arginine and	38	0.42895	2	0.066457	1.1775	1	0.3489	0.01212
Valine, leuci	40	0.45153	2	0.072772	1.138	1	0.35718	0
Thiamine me	7	0.079017	1	0.076539	1.1161	1	0.35718	0
Taurine and	8	0.090305	1	0.087011	1.0604	1	0.38468	0
Ubiquinone a	9	0.10159	1	0.097373	1.0116	1	0.40896	0
Arginine bio	14	0.15803	1	0.14754	0.83109	1	0.59015	0
Butanoate m	15	0.16932	1	0.15725	0.8034	1	0.60042	0.11111
Sphingolipid	21	0.23705	1	0.21338	0.67085	1	0.74682	0
beta-Alanine	21	0.23705	1	0.21338	0.67085	1	0.74682	0.39925
Glycolysis /	26	0.29349	1	0.25744	0.58932	1	0.865	0.10044
Porphyrin an	30	0.33865	1	0.29101	0.53609	1	0.94018	0
Leukotriene	39	0.44024	1	0.36139	0.44203	1	1	0
Primary bile	46	0.51926	1	0.41152	0.38561	1	1	0.02239
