# Supplementary material for: Integrated Serum Metabolomics and Network Pharmacology to Reveal the Interventional Effects of Quzhi Decoction against Osteoarthritis Pain
Source: Int J Anal Chem. 2022 Aug 12;2022:9116175. doi: 10.1155/2022/9116175 (PMC9391123; doi:10.1155/2022/9116175)
Supplement: Supplementary Materials — Supplementary Material 1: self-defined synovial pathological scores. Supplementary Material 2: serum metabolites and metabolic pathway. Supplementary Material 3: the common gene of Quzhi decoction in the treatment of OA. [file 9116175.f1.zip › Supplementary Material 3.pdf]

The common gene of Quzhi decoction in the treatment of OA.

Disease targets			Drug targets	Overlapped targets
TGFA	CDC5L	NAA30	PTGS1	PPARA
LRCH1	MIR210	ADCK2	CHRM3	IL6
PPARA	RAC1	OTULIN	CHRM1	PTGS2
TLR2	CNR1	SHROOM2	SCN5A	PON1
TAC1	EIF6	MESP2	CHRM5	MMP2
IL6	SIRT6	RIPPLY3	PTGS2	MMP1
ANKRD6	MRC1	IGLL5	HTR3A	IL1A
BMP5	LRP6	RIPPLY1	CHRM4	IL1B
PTGS2	EIF2AK3	LRMDA	RXRA	PLAU
KHDRBS1	S100B	KRTAP4-11	OPRD1	MMP3
PON1	CSGALNACT1	LOC100130744	ADRA1A	MMP9
TACR1	MYD88	OA3	CHRM2	MAPK8
XCL2	COL27A1	OASD	ADRA1B	AR
MMP2	CBS	MRC2	SLC6A3	ESR1
MMP1	ZMPSTE24	POSTN	ADRB2	SERPINE1
ADAM12	NOS3	NTRK1	ADRA1D	PTGS1
NME8	AIP	SMN1	OPRM1	BCL2
THRSP	GHRL	ITLN1	GABRA1	THBD
TNF	BMPR1A	NT5E	NCOA2	PPARG
LINC01006	PTGER2	ENO2	NCOA1	AKR1C3
IL1A	FGF1	FOXD2-AS1	SLC6A4	ALOX5
BTNL2	HYAL2	CRYAB	PGR	AKR1B1
MIR155	NR1D1	PBRM1	NR3C2	PPARD
PEPD	CAMP	DEFB4A	ACHE	IL10
KL	FGF18	IL12B	DRD2	CRP
ASPN	NOS1	SHBG	ESR1	CXCL8
EPAS1	CHRDL2	IL1RL1	AR	COL1A1
ACE	MIR145	SETD2	PPARG	RUNX2
PCSK6	DUS4L	OPRD1	AKR1B1	COL3A1
TRPV1	PIK3C2A	ATP7B	PRSS1	CCL2
IL1B	PTN	MIR34A	RELA	VEGFA
COL6A4P1	CXCR2	THRA	EGFR	SPP1
TNFRSF1B	CS	RNASE3	AKT1	IFNG
TGFB1	TNFAIP3	IL34	VEGFA	IL4
COX2	CFLAR	OLR1	CCND1	ICAM1
SOCS3	NCOR2	NUCB2	BCL2L1	NOS2
PLAU	GZMA	IGFBP1	CDKN1A	VCAM1
SMAD3	NCOA3	TLR8	CASP9	IL2
TLR9	TREX1	EN1	MMP2	CCND1
IL1RN	HDAC9	DIO2	MMP9	STAT1
GDF5	OPRK1	GPR22	MAPK1	JUN
ACAN	ITGA1	CTSD	IL10	TYR
RHOB	HRH4	CXCL11	RB1	IGFBP3
TNFSF11	SOX4	LOC101928222	TNFSF15	MAPK14
COL6A4P2	PPP3CA	DPP4	JUN	FOS
MMP3	MECP2	MIR671	IL6	CASP3
MMP9	NGFR	PNOC	CASP3	CD40LG
GLIS3	MIR22	CCL22	TP63	AKT1
NFKB1	HMMR	IFNB1	NFKBIA	MAPK1
AGER	ACTG1	MIR27A	TOP1	CAT
BAG6	PTK2B	FGA	MDM2	EGF
TNFRSF11B	PGF	MIR130A	APP	CXCL10
D1S111	SERPINA4	NR1I3	MMP1	STAT3

BCL9	CRLF1	ITGB3	PCNA	SELE
MAPK8	AHSG	PCSK9	ERBB2	IGF2
TNFRSF1A	PAX7	PRKCA	HMOX1	PTGES
POU5F1	IGFBP5	CCN4	CASP7	OPRM1
TLR3	FGF21	HMOX1	ICAM1	F3
BUD31	MIR206	CYBA	MCL1	MPO
TENT5A	PREP	GHRHR	BIRC5	NFKBIA
HFE	USP8	HRH1	IL2	MYC
PITX1	DEAF1	SOX11	CCNB1	CDKN1A
ZNF410	SERPINA1	PLAT	TYR	CXCL2
SLC22A3	KDM6A	NOG	IFNG	MTOR
LPAR1	CIRBP	INF2	IL4	SOD1
ALDH1A2	CCR3	SLC26A3	TOP2A	KDR
CD40	HAS2	MFAP5	GSTP1	CALCR
COL2A1	TSLP	LTBP4	SLC2A4	HIF1A
VDR	FNDC5	MIR221	INSR	MDM2
CALCA	THBS4	XDH	CD40LG	F7
CCR2	LOXL3	CYP19A1	PTGES	RELA
BDKRB2	IL1RL2	KDM1A	NUF2	ESR2
CDX2	NFE2L2	JAM3	ADCY2	MET
IL16	GANAB	IL18BP	MET	BAX
MAGI1	MAF	MVK	CCNA2	NR3C1
AR	CSK	CD19	ADRA2A	GJA1
COMP	CRBN	PAX2	ADRA2C	PLA2G4A
MOK	CCDC54	NPR2	NOS2	PLAT
COL9A2	ERG	SLC9A3R1	ESR2	APP
PCYT1A	WWP2	COL4A3	MAPK14	CASP8
PDE3B	MIR300	COL4A4	GSK3B	SLPI
TNFAIP6	CHKA	SGSH	NR3C1	GSTM1
IL18	IFRD1	RPS26	KCNH2	EGFR
SFRP1	LOXL2	RARRES2	STAT3	OPRD1
TCP1	IGFBP2	GPI	EDN3	CTSD
ESR1	MIR98	HSP90AA1	CHEK1	CXCL11
PRG4	PARP1	ADAMTS15	CHRNA2	NR1I3
TGFBR1	POMGNT2	BSG	DRD5	ITGB3
MIR21	CCL8	RECK	F7	PRKCA
MIR223	SQSTM1	TREM1	SLC6A2	HMOX1
SERPINE1	GRN	MMP17	ADRA2B	EIF6
SOST	MCAM	MSTN	GABRG3	NOS3
PDPK1	PRKAR2B	EP300	GABRE	PPP3CA
CA2	BCAP29	GSR	BCL2	HAS2
CA3	MIR128-1	IL22RA1	FOS	NFE2L2
CDH11	HPBP	CHIT1	AHSA1	PARP1
PTGS1	GLI1	JAK1	FASN	ERBB2
BCL2	ADAMTS1	MAP3K8	EDNRA	E2F1
THBD	THPO	FCGR2B	CYP3A4	CASP9
FABP2	XRCC4	SRF	CYP1A2	CAV1
PPARG	CHADL	LECT2	MYC	BIRC5
CFTR	DEFB103B	IL26	CYP1A1	CYP3A4
GP1BA	DNMT3A	VCAN	NR1I2	GSK3B
S100A7	SERPINB5	HDAC4	NPM1	CHUK
PLA2G2A	SLC2A1	DCSTAMP	ECE1	AHR
PTGR2	CCR4	VIM	PARP4	NPM1
GLO1	GH1	ADCY3	CALCR	IRF1
PTGDR2	CSTA	TYRP1	ITGB3	PGR
AKR1C3	CXADR	NCAM1	BAX	HSPB1

CXCR1	CXCL13	GPT2	CASP8	GSTP1
ALOX5	CREB1	SLC26A5	PRKCA	TP63
KCNQ2	FOXP4	SLC6A11	PON1	ADRB2
KCNQ3	SHROOM1	SRSF7	MAP2	PCNA
pab	AKNA	SLC26A1	ADH1C	CCNB1
AKR1B1	ZSWIM6	SLC26A8	PLAU	CASP7
MAPK3	ANP32A	ARF3	LTA4H	
PPARD	PTPRD	SLC38A4	MAOB	
AKR1B10	RPL29	SLC7A3	MAOA	
MATN3	AQP1	RAP2B	CTRB1	
COL9A1	MIR203A	SLC26A6	ADRB1	
MMP13	MIR29B1	HPS6	CXCL8	
COL9A3	TWIST1	GGA3	PRKCB	
SLC26A2	MIR454	GGCT	IGF2	
COL11A2	MIR885	TTC3	GSTM1	
FRZB	FCGR1A	SUGCT	GSTM2	
FBN1	TH	TAAR9	GSTA1	
DDR2	KERA	GGT2	GSTA2	
TRPV4	JAK2	MIR222	GRIA2	
COL10A1	ALOX15	GGTLC3	MMP3	
TGFB3	IDH1	TMPO-AS1	EIF6	
COL11A1	IDH2	TGFB2-AS1	EGF	
UFSP2	GNA11	TGFB2-OT1	ELK1	
IL10	TCIRG1	LOC101448202	POR	
TRAPPC2	OSTM1	ENSG00000259039	ODC1	
CANT1	IL1R2	IL4R	RAF1	
TGFB2	STAT6	TRPC6	SOD1	
CCN6	TSPAN4	SIGLEC1	HIF1A	
TGFBR2	FOXO3	PRRC2A	STAT1	
CRP	CAPN2	APP	RUNX1T1	
CXCL8	SOX6	DOT1L	ACACA	
IL17A	SPON1	AK1	CAV1	
TIMP1	MUTYH	PPP1CC	F3	
GALNS	CD58	FCGR2A	GJA1	
FGFR3	VTN	BST1	IL1B	
COL1A2	SOD3	LTB	CCL2	
ALB	LGALS1	HLA-DRB5	SELE	
BGLAP	HDAC7	IFNA1	VCAM1	
COL1A1	OLIG3	BTLA	PTGER3	
RUNX2	ITGAX	HTR2A	DUOX2	
COL5A2	ERBB2	CD38	NOS3	
ADAMTS5	CCL27	TRAF3IP2	HSPB1	
KIF22	TBX5	IL10RA	SULT1E1	
ADAMTS4	UCN	MIR29A	MGAM	
IL1R1	AMBP	PRPS1	CYP1B1	
SOX9	CA1	GNAQ	PLAT	
CHI3L1	E2F1	ASS1	THBD	
COL5A1	CILP2	WT1	SERPINE1	
CILP	XIAP	HLA-DRA	COL1A1	
DCN	IL36A	SLC19A1	ALOX5	
AEBP1	IL33	SH2D1A	IL1A	
MATN1	ITCH	DHODH	MPO	
TP53	CASP9	NPHS1	NCF1	
FBN2	JAG1	ACTN4	ABCG2	
NOD2	CAV1	TAP2	HAS2	
PTH1R	PDE4A	ANK3	NFE2L2	

HLA-B	MIR24-1	TAPBP	NQO1
LOC109461476	HTT	APBB1	PARP1
ANKH	MAP1LC3A	HLA-DMB	AHR
COL3A1	NLRP12	MICB	PSMD3
FLNA	MMP16	HLA-DOA	COL3A1
MEFV	TCF7L1	NPHS2	CXCL11
GNAS	VCP	HLA-DMA	CXCL2
PHEX	PIK3R1	CELSR3	DCAF5
MGP	WNT7A	PDLIM5	NR1I3
FLG	AZU1	HEPH	CHEK2
XYLT1	MFGE8	DDX39B	CLDN4
CCL2	DDIT3	CCRL2	PPARA
LTA	PDPN	LDB1	PPARD
IHH	ITGA6	LDB2	HSF1
MIR140	ANXA2	TAGAP	CRP
LMX1B	SP7	FXYD1	CXCL10
VEGFA	CCL21	LRRK1	CHUK
CLCN7	PSAP	KRT86	SPP1
HAPLN1	FMR1	IGDCC3	RUNX2
CTSB	REN	SIAE	RASSF1
IGF1	NEAT1	ZNF354A	E2F1
NLRP3	BIRC5	SUPT20H	E2F2
ADAMTS14	CEBPB	IFRD2	ACPP
NLRP1	C3	PPFIA3	CTSD
TLR4	CMA1	TBL1X	IGFBP3
SPP1	CD63	CLEC16A	IRF1
MCF2L	TARDBP	LIN54	ERBB3
ERCC6	CDKN1B	CEP350	DIO1
KIF7	STAT5B	ZNF334	PCOLCE
ACP5	S100A4	USP50	NPEPPS
MIR146A	HDAC8	PDF	HK2
HRAS	PDCD5	FRG2C	RASA1
BMP2	ENPP2	FBXL19-AS1	IKBKB
IFNG	UTS2	SNHG28	MAPK8
PTH	CSGALNACT2	PCAT1	PPP3CA
CD36	SMURF2	SNHG29	AKR1C3
H19	CYP3A4	RNU105C	SLPI
PTHLH	WNT3A	HCG14	RXRB
SCN9A	CNR2	CCAT1	KDR
TMSB4X	DNTT	LOC106146143	CAT
MMP14	VIM2P	LOC106146144	PLA2G4A
IL23R	CYP2D6	LOC106146150	ABCC2
PTPN22	ARNTL	LOC106146152	MTOR
IL4	UGDH	LEPQTL1	PKIA
MMP8	F13A1	DDB1	
TRPS1	GSK3B	EPO	
LEP	FOXO1	CORIN	
ALPL	DANCR	APOB	
IL6ST	MIR381	NPY	
HPGD	PRKN	PARD3B	
FKBP14	CPNE1	CCL13	
ICAM1	RBM39	MMP7	
SMAD2	GGT7	TSBP1-AS1	
CCL5	NCOA6	TGM2	
ADAM10	RBM12	EMD	
HLA-DRB1	ERGIC3	SUMF1	

HOTAIR	KIF12	CLEC3B
SEMA3A	AAR2	IFIH1
PACERR	CNBD2	HERC2
DYM	MMP24OS	GUSB
GAS5	piR-46368	KCNJ11
SMAD4	piR-57133-366	ITGB1
IL15	KCNA2	ATN1
HGD	DDX58	UQCC1
PSTPIP1	ELP1	GLT8D1
LMNA	IFNL1	SREBF2
PADI4	CHRNA7	KNG1
NOS2	XIST	CASP8
CCL3	SNORD19	CDK6
KCNK15-AS1	TPI1	ACVR1
CASR	TERC	GK
UCMA	GSTT1	FAH
ENPP1	TRPV6	PAX1
F9	L13304-025	CLCN5
GBA2	L13715-020	TSR2
MEG3	AB372731	SUPT3H
IL1RAPL2	MC1R	LRP1
PAPSS2	ERVK-18	ACTC1
PRKAR1A	POLG	PRL
VCAM1	ATR	LUM
PTPN11	MIR146B	BGN
TRAPPC2B	BTK	OPTC
TIMP2	CCL17	SNAI1
SERPINA3	HBEGF	KIR2DL1
BMP7	MSH6	KIR2DS4
S100A9	AREG	INHBA
IL2	ADIPOR1	MAP2K1
CCND1	MAP2K6	SOX5
HLA-DQB1	DAG1	SLPI
CXCL12	TPSAB1	CHAT
CPT2	HRH2	IL1RAP
UFC1	MIR320A	CCR7
STAT1	LNCRNA-ATB	RIPK4
CSF1	THBS2	GDF6
ACTA2	RNASEH2C	RSPO2
EFEMP2	CMKLR1	GSTM1
KCNJ5	IL17D	NR1H4
FN1	GC	CDK1
CTSK	FIP1L1	ARL13B
HMGB1	SMPD1	CST3
TTR	G6PD	SCARB2
B3GAT3	IL27	HAVCR2
PCGEM1	MIR125B1	EGFR
FAS	MIR125B2	IRAK3
CXCL1	MIR675	GPBAR1
ATP7A	NOD1	CASC3
PMS2P2	STAB1	MAP2K2
ADIPOQ	HMGB2	PECAM1
RETN	HPD	CDH2
KCTD13	SCG2	WNT5A
JUN	PPARGC1A	SERPINF2
MYH11	MMP28	KCNQ1

CCR6	NOX1	PRKDC
SMAD6	ADAMTS7	SNCA
SLC2A10	PPBP	DBH
OSM	GRP	ILK
MIF	FURIN	STS
GHR	MIR148A	GNAI3
IL7	MIR365A	ADAMTS13
DKK1	KIT	BIRC2
RET	RRM2B	CHN1
TLR5	SP1	FDPS
TYR	PTGER4	CACNA1F
NGF	MIR488	PSMB5
IL23A	P4HTM	GALC
F8	CAMK2G	M6PR
CIITA	DNMT1	CLCN4
CD44	ITIH1	HCCS
IL13	CEACAM4	GPR143
IL6R	GALE	OCA2
ELN	NFKB2	MEOX1
IGFBP3	GLA	GDF3
GBA	HOXD10	SLC24A5
FGF2	CLEC4A	SUCLG2
F2	GRPR	MLANA
MAPK14	ANGPT2	SLC45A2
TNFRSF11A	DEFB104A	TBX6
LRP5	DEFB104B	HPS4
MEN1	MIR195	GPRC6A
FOS	ADAM8	FRMD7
CASP3	MIR31	MYO18B
HSPD1	HNRNPC	CABP4
ELANE	GRIN2B	SPECC1L
BRCA2	HLA-G	HES7
SPDT	CCL7	RIPPLY2
PES1	BAG1	MIR92A1
FMOD	DPP7	AGRN
CHST11	MIR9-3	ALOX5AP
SFRP4	MIR9-2	HAS3
CD40LG	MAPK10	TYMP
FOXP3	LOC106728418	CXCL16
H2AC18	HCFC1	PDGFB
AKT1	CHUK	CFP
MAPK1	SLC39A4	TNKS
FASLG	GRB2	CA10
TIMP3	CLCNKB	TRIB3
IBSP	BSND	MIR33A
CCN2	CRTC1	RPS6KB1
S100A12	CSMD1	HEXA
ADAMTSL1	TMEM167A	CPOX
KRAS	SCARNA18	CALR
IL18R1	ADAMTS8	BPI
ADAM17	ADIPOR2	CYBB
EZH2	PTGES3	ROR2
IL11	MIR139	CKM
CCL4	TERT	MADCAM1
CDK4	ITGAV	C4BPA
ATIC	DUSP1	ANG

MIR132	FAAH	VEGFC
NRAS	KLF5	EIF4EBP1
CCL18	CSPG4	MYOM2
CAT	ZFP36L1	TTF2
IL2RA	LPA	TFDP1
HP	TSC22D3	TIA1
CCL20	NID2	OMD
EGF	TUT7	MMP19
CTLA4	MIR16-1	SCARB1
HSPA5	MIR16-2	PDGFA
CXCL10	MIR20A	SMAD1
FGF23	ADM	TIE1
CYP27B1	CENPJ	DHRS3
STAT3	MIR105-1	PDYN
BRAF	ATP6V0E2	OXT
SELE	MIR320C1	ACLY
PLOD1	AHR	SEMA4A
GAPDH	HIF3A	OPRL1
CSF2	HBP1	HDC
B2M	PRNCR1	PDGFRB
CXCR3	NPM1	EPHB4
IGF2	SOCS2	RHOA
EFEMP1	CRADD	CNTF
NOTCH1	AP3B1	FSHR
MTHFR	ITIH4	MC2R
EXT1	MMP24	CXCR6
PLAUR	NFAT5	RBPJ
CCND2	RASGRP3	AQP9
ERCC1	PUF60	SFMBT2
LTBP2	CYP2A13	CNMD
BANF1	DYNC1I1	IL12A
KMT2D	NEK4	RAG1
ARID1B	SCRIB	SLC2A3
SERPINC1	CEP250	DEFB1
VIP	CEACAM3	DEFA5
MIR150	ABCB9	DEFA3
RELN	B9D2	DEFA6
CX3CL1	SMG6	CYP2C9
PTGES	RAD54L2	CD247
DMP1	DDX10	XYLT2
BDNF	RPGRIP1L	EFNB2
BMP6	CDK2AP1	CD9
COMT	AGAP1	TNFRSF12A
DSPP	AAGAB	CD84
OPRM1	CHD9	SMAD5
SLC39A8	FRMD4A	CHRD
PTEN	TIPIN	NR1H2
F3	KCNIP4	EFNB1
SLC22A4	HNRNPUL1	ACD
INS	NDST4	ANXA6
CD244	PARP10	C5
NFKBIL1	POGK	ACSL4
CSF1R	PPM1H	SECISBP2
ARSH	ISLR	LTF
MIA2	TMEM91	PNLIP
FBLN2	MOB3B	PITX2

CD4	EXOSC4	CBFB
CTNNB1	LRRN1	DLX5
ALPP	TAS1R2	PHOSPHO1
COG5	ISLR2	FAM215A
HLA-DQA2	SLBP	IFI27
SAA1	KIAA1217	PTK2
HLA-DQA1	RWDD2B	THY1
CXCL9	SBNO1	DLG4
CD80	NT5DC2	DDAH2
SERPINH1	RRP9	GSN
CRYAA	BBX	IGFBP4
SPG7	THSD7B	MIR199A2
MICA	MPHOSPH9	FLT4
ITGAM	CCDC33	ZAP70
PRTN3	ABHD14B	SUMO1
MPO	EPPK1	TCF7L2
SLC40A1	ZC3H3	GRK6
GPR101	ZWILCH	CITED2
MRAP	NRBP2	VIPR2
THBS1	GALNT18	INHA
FGFR2	IQCH	KDM6B
ITGB2	POLR2M	NRP2
FBLN1	SPATC1	FZD10
TNR	CEACAM21	ALCAM
PIK3CG	CCDC97	CDO1
ADAMTS3	FAM53A	PCM1
IL22	FAM83C	SEMA3F
MMP10	BTNL8	ACKR3
B3GALT6	ZBED5	TEP1
LPL	FAM98A	TERF2
FSTL1	C11orf87	CDKN3
F5	EQTN	C1D
RMRP	PLPP6	UCN3
C1S	TMEM61	HECTD2
C1R	C8orf82	GCNT4
FCGR3A	MS4A13	GALNT15
IGF1R	SMIM4	PCOLCE2
CCR5	STIMATE	UCN2
MMP12	MANSC4	RBIS
ZNF687	MIR661	MIR19B1
IL17RA	SNORD55	MIR519B
CASP1	FAM83C-AS1	MIR608
CP	MIR1289-1	MIR558
ANXA5	DUBR	MIR602
GPR33	ATP2B1-AS1	STK11
ACVRL1	ZC3H11B	OGN
AP3D1	LYPLAL1-AS1	PCNA
HMGCR	LSP1P3	HSPB2
EXT2	ENSG00000255224	RFT1
MIR9-1	SBNO1-AS1	NELFA
CTSL	ROCR	EEF2
IDS	ENSG00000196295	ALDOA
LIG4	LOC729254	IRS1
ERCC8	LINC02399	CCNB1
HAS1	ENSG0000234091	CD47
PLOD2	RNA5SP301	C1QTNF3

FLT1	MTND4P14	H6PD
TGFBR3	ENSG00000228322	GCFC2
SLC34A3	ENSG00000258216	DCC
SELP	ENSG00000259618	REST
ANXA1	ENSG00000261384	COPS5
CD86	ENSG00000270195	MC5R
ICOSLG	LINC02742	SFRP5
SMAP2	ENSG00000207189	ACKR1
LIF	ENSG00000257042	SHC1
SIK3	LINC02752	FPR2
IL2RB	ENSG00000260773	HPSE
FGFR1	ENSG00000250453	SIRPA
STAT4	MRPS33P3	P2RX1
NFKBIA	RNA5SP349	PADI3
COX5A	lnc-GLIS3-2	CDH13
SIRT1	ENSG00000223727	BAIAP2L1
HSPG2	BRWD1P2	PRTG
SST	RF00017-5091	SNX19
TGIF1	lnc-IQCH-7	TALDO1P1
TRAPPC3	ENSG00000238232	PRKCD
TRAPPC12	LOC105372401	GNB3
OCRL	CNN2P10	ADAMTS2
SELL	lnc-DYNC1I1-4	PLA2G6
CDKN2A	lnc-FAM83C-2	PTPN2
TNXB	lnc-HFM1-3	MYCN
TMPO	NONHSAG011935.2	HMGA1
GLB1	lnc-TACC3-2	C1QA
TNFSF13B	piR-50208	C1QB
HLA-DPB1	RF00017-5093	C1QC
CD28	lnc-ATP2B1-1	CENPE
MYC	lnc-PRPF18-2	RAG2
CDKN1A	piR-39858-250	AGTR2
MATN4	ENSG00000285914	PHOX2B
REL	RF00017-7371	GNAI1
SPARC	RF00017-7373	A2M
ADAMTS9	RF00017-7404	PPM1D
TEK	lnc-SOCS2-6	KLK3
ABCB1	lnc-B9D2-2	P2RY2
IRF5	lnc-CD47-7	SLC20A1
NF1	lnc-STAB1-1	ABCB7
CXCL5	ENSG00000277749	TXNDC5
CXCL2	lnc-PLEC-4	IFNGR1
APOH	ENSG00000273254	TXNRD2
PLEC	lnc-LYPLAL1-11	HSPA1A
MTOR	piR-38533	PTPN1
CD163	lnc-NT5DC2-1	CASP7
LTBP1	piR-30534	TRPC5
P4HB	lnc-GGCT-3	KCNN1
ENG	HSALNG0005172	MAT2B
SOD1	piR-43107-151	SLC16A4
CD79A	lnc-KLHL42-2	TP53AIP1
TRAPPC9	RF00951-040	SERPINF1
MIA3	piR-52471-007	CLTC
TRAPPC1	LOC100288365	NTRK3
TRAPPC4	HSALNG0059706	CASP10
TRAPPC10	NONHSAG001562.2	AXIN2

TRAPPC6A	piR-36756-038	PDE8B
TRAPPC2L	lnc-CTR9-7	AIMP1
TRAPPC5	RF00100-076	COL4A5
TRAPPC8	LOC649024	DSE
TNFSF13	lnc-CDC5L-7	KRT18
CYCS	lnc-RPGRIP1L-2	GAST
MIR335	ENSG00000288096	EGLN1
SDHA	ENSG00000286910	S1PR2
IL17F	piR-32559-090	PLAA
CTSG	RF00017-596	PPARGC1B
MALAT1	ENSG00000283517	NAA80
F10	piR-57137-006	OS4
TLR10	RF00017-3159	OS6
ANGPT1	lnc-ADAM10-10	CCAL1
MIRLET7E	ENSG00000285577	EDM5
LIFR	RF00017-1169	FRZB1
HLA-A	ENSG00000256378	GOA1
PRODH	lnc-PBRM1-2	MSK16
SLC35D1	SMC4P1	OS5
KDR	LOC105369890	PLAP1
TRAF6	ENSG00000254957	DUPANS
SDC1	RF00004-011	AGC1
IL32	LOC101929770	CDMP1
HSP90B1	ENSG00000286417	HOA
LOX	RF00017-5581	SRFP3
CXCR4	piR-56368	SEDK
SLC11A1	piR-56497-029	BDA1C
MUC1	ENSG00000274075	OS3
STEAP4	LOC105373943	CSPG1
MIR199A1	LOC105377720	SYNS2
CALCR	LOC105377721	OS2
PRKG1	piR-56497-036	OS1
CCN1	ENSG00000283601	SSOAOD
FTO	ADAM15	SYM1B
HIF1A	C5AR1	OAP
SHOX	SLC23A2	TINAGL1
MIR17	PRLR	SLEN3
CXCR5	DIO3	SLEN2
U2AF1	S100A11	SLEN1
PRDM10	LGALS9	TINAG
BMP4	ALK	GPNMB
EDEM2	GNS	5-LOX
SOD2	GZMB	MMP-2
ITGA4	KITLG	TrkA
VWF	CABIN1	COX-1
CD69	CYTL1	FXR
TRAF3	TNFRSF6B	MAPK12
F2RL1	BMP2K	COX-2
ARSA	EIF1AD	COX
ITGAL	USP33	MMP-13
THBS3	LTBP3	IKKB
SLC34A1	IL24	BDKRB1
CSF3	IL20	MMP-3
CD8A	ATF3	NaC
GALNT3	MME	p38
UCA1	XPA	MMP

HNF4A	RFC1	ALOX
FOLR2	HMGA2	NSD2
CAST	ANGPTL4	NAGLU
GNL3	TNFSF12	SYVN1
GFPT1	ICAM3	IGF2R
OTOG	SERPINE2	CRH
TRPM6	IL18RAP	CCL11
KDM4C	TCF4	GJA1
CAPZB	MELTF-AS1	DSP
POMC	ULK1	TNIP1
SOCS1	ITGB7	PLA2G4A
SYK	PKM	PVT1
TTN	GPX3	ZFAS1
PROCR	DDIT4	IL9
ENO1	EIF4G2	SLC17A5
LRRK2	KLK11	HGF
OLAH	CD160	SLC22A12
PTPRC	CRTAC1	ERAP1
NAMPT	CORT	TNFRSF8
S100A8	HSH2D	FCAR
CANX	MIR138-1	NPSR1
FGF7	MIR138-2	CARD8
CD68	MIR486-1	MIR99B
PF4	IRF3	CALM1
NR4A2	RNPC3	EEF1A2
HSPA4	CSN1S1	MIR193B
TNFRSF13C	SLC3A2	SMAD7
MBL2	IRF1	HNRNPA2B1
CD14	IL17B	MAPT
PYCARD	BMP3	ABT1
MACIR	PGR	AMPD2
FBLN5	HSPB1	LOXL1
ARSB	ACER3	ITGA5
APOA1	ABCA1	CD151
TRIM2	NR1H3	NTF4
AGTR1	P2RX7	ENPP3
FCRL3	FST	SRD5A1
LACC1	PRKAA1	CYSLTR1
HNRNPL	EEF1A1	BNIP3
STRC	MAP2K3	ITGAE
CEACAM16	MIR29B2	CD83
NAA50	MIR29C	RNASE7
CX3CR1	TF	NTRK2
IDUA	WRN	PDGFRA
SYNE1	MMP11	ALPI
MDM2	NTF3	ADD1
MIR30A	TYK2	IL15RA
HTRA1	CD59	SETDB1
IL19	GSTP1	SCUBE1
F7	TP63	BMP1
SLC26A4	KEAP1	BAX
MAFB	PDE5A	BLK
TNFSF10	RACK1	TFRC
MYLK	MIR181C	RELB
FAM20C	MKX	TMEM43
CXCL6	JUNB	H3-2

RELA	TFAP2A	APOE
SAA4	LAMA4	GPT
ESR2	TNFRSF18	NKX3-2
TBX4	KIR3DL1	PDCD1
TLR1	TRAIP	IL37
PKD1	NLRP6	TRAF1
GREM1	KIR2DL3	MITF
TLR7	HYAL1	FMO1
GGT1	SPHK2	PIGB
MEPE	PADI2	SMYD1
LEPR	NRP1	ANKAR
IL21	BMPR1B	NEMP2
LBR	HPRT1	LOC102723692
MIR15A	LPIN2	LOC113939944
PSMB9	ORAI1	NR3C1
RPE65	TNFSF14	CCR1
PLCE1	GRIN2D	TNC
CD2AP	PRNP	IL3
ZNF23	ITGA2B	CR1
BLOC1S1	F2R	IL5
TMEM222	PSMB8	CFI
RDM1	IKBKG	LGALS3
EMSY	TNFRSF13B	TUG1
OR1L1	MTR	GNRH1
MIR142	TAT	AP2S1
MIR196A1	SLC29A3	TRH
FCGR3B	HAMP	PLEKHM1
CD34	MTRR	GCM2
EDN1	ATP6V0A2	LEMD3
MET	CREB3L1	SNX10
ITGA2	ADRB2	ALX4
EZR	PIK3CB	KIAA0319L
CGAS	DLL1	HGSNAT
HOTTIP	LGR5	HJV
SULF1	LGR4	BHD
VIPR1	FUT4	ATF4
CALCRL	CCAR2	P4HA2
ENTPD1	SETD1A	
